# Review on kidney diseases: types, treatment and potential of stem cell therapy

**DOI:** 10.1186/s41100-023-00475-2

**Published:** 2023-04-27

**Authors:** Jaspreet Singh, Sanjeev Singh

**Affiliations:** School of Bioengineering & Biosciences, Lovely Professional University, 15935, Block 56, Room No 202, Phagwara, Punjab 144411 India

**Keywords:** Acute kidney injury, Chronic kidney diseases, Stem cells, Medicine for kidney diseases

## Abstract

Renal disorders are an emerging global public health issue with a higher growth rate despite progress in supportive therapies. In order to find more promising treatments to stimulate renal repair, stem cell-based technology has been proposed as a potentially therapeutic option. The self-renewal and proliferative nature of stem cells raised the hope to fight against various diseases. Similarly, it opens a new path for the treatment and repair of damaged renal cells. This review focuses on the types of renal diseases; acute and chronic kidney disease—their statistical data, and the conventional drugs used for treatment. It includes the possible stem cell therapy mechanisms involved and outcomes recorded so far, the limitations of using these regenerative medicines, and the progressive improvement in stem cell therapy by adopting approaches like PiggyBac, Sleeping Beauty, and the Sendai virus. Specifically, about the paracrine activities of amniotic fluid stem cells, renal stem cells, embryonic stem cells, mesenchymal stem cell, induced pluripotent stem cells as well as other stem cells.

## Background

Kidneys are an overly sensitive organ with restricted regenerative efficiency with respect to other vital organs. Along with cardiovascular disease, diabetes, and hypertension; renal disorders are one of the most rapidly growing health issues around the globe. So, the research on renal tissue repair and regeneration has ignited a new field of study that focuses on various regenerative options. Because in order to avoid renal injury, the recovery of affected cells is as significant as preventing its progression towards end stage renal disease [1]. So as a new therapeutic approach, stem cells are under research with a focus on reducing the burden of several kidney diseases.

According to the 2018 survey of the National Kidney Foundation, around 37 million people in the USA are affected by kidney failure, while almost 750,000 patients endure kidney failure annually, making it the leading cause of death among the US population [2]. In India, as per the report of 2018, the average capital spent by a kidney patient on each haemodialysis session is around USD64. The Catastrophic health expenditure for a patient with two dialysis sessions per week was estimated as 38.1 per cent which increases to 52 per cent for patient undergoing haemodialysis thrice a week [3].

Glomerular filtration rate (GFR) is the most accessible guide for normal kidney function, which rises to the aggregate sum of fluid filtered through the entirety of the working nephrons per unit of time [4]. It is predicted as the clearance of the exogenous marker after a solitary bolus infusion of marker and may be present in plasma as well as urine concentration estimations or in plasma estimations alone [5].

The GFR examination and albuminuria, a marker of kidney damage, are both used by nephrologists to categorise kidney conditions into two categories: acute and chronic kidney disorders. Moreover, it was found that these two are firmly correlated, which means acute renal failure can lead to chronic type and vice versa [6].

1. Acute Kidney Injury (AKI)

An unexpected decrease in urine output followed by a rise in serum creatinine concentration, including an inability to excrete waste, keep-up electrolytes, and maintain water balance, is termed acute kidney injury (AKI) or acute renal failure. According to AKI Network criteria, AKI is classified in multiple stages, which initiates as an arbitrary increment in serum creatinine levels of at least 26.5 mol/L (rise of almost 1.5–2.0-fold from the reference line) with urine output less than 0.5 mL/kg/h for more than 6 h and is used as a mark for a patient to be classified under stage 1 or the initial stage of AKI. Further, for stage 2, serum creatinine levels should be around 2.0–3.0-fold above the reference line with urine output below 0.5 mL/kg/h for over 12 h. This lasts till 3rd stage with serum creatinine level above 354 mol/L with urine output below 0.3 mL/kg/h for a complete day. In some cases, patients who initiated renal replacement therapy are directly classified under stage 3 AKI [7].

A few investigations have demonstrated a direct relation between AKI and expanded risk for death [8]. Deaths because of renal failure among 15- to 69-year-olds were seen as 2.9 per cent of all deaths in 2010–13, which is an increment of half from 2001–03. The announced extent of renal failure deaths is close to the global burden of disease estimate for 2015 of 3.04 per cent, up from 1.94 per cent in 2000 [9] and in addition to this, each year around 1.7 million deaths are recorded due to AKI [10]. It was identified that a huge extent of renal patients in the growing world, including India, die without renal replacement therapy [11].

For the investigation of its cause, kidney injury is generally classified into three categories: prerenal, renal, and postrenal structures. Prerenal AKI is caused by obstructed blood flow towards the kidneys because of reduced blood volume and factors restricting blood flow inside the kidney. Renal AKI is because of destruction of renal parenchyma, whereas the last one, postrenal AKI is the because of obstacle in urinary tract. The foremost recognised reasons for AKI are, as often as possible, related to renal ischemia, infection, and nephrotoxic medications [12].

Prior to the rise in serum creatinine level, IL-18 (Interleukin-18), NGAL (neutrophil gelatinase–related lipocalin), and KIM-1 (Kidney Injury Molecule-1) had been used as biomarkers to predict the potential risk of AKI [13]. However, the possible biomarkers to diagnose the risk factors for AKI are being distinguished and assessed in clinical investigations. Despite some advancement, there are no guaranteed biomarkers that could be utilised to classify patients based on the risk factor [6].

As indicated by certain findings, renal ischemia–reperfusion injury has been found to be one of the most broadly perceived explanations behind AKI [14]. Not only I/R injury but also the patients having cardiovascular disorder and increased intra-abdominal pressure, ends up by restricted blood flow through renal arteries further followed by AKI or debilitation of the already compromised renal function [15]. Apart from I/R injury, diabetes mellitus, age, and genetic factors; the pre-existing chronic kidney disorder is one of the most usual reasons for AKI which can multiply the risk to multiple fold with respect to the patients with no initial sign of chronic kidney disease [16].

2. Chronic Kidney Disease (CKD)

According to National Kidney Foundation-Kidney Disease Outcomes Quality Initiative (NKF-KDOQI) and National Institute for Health and Care Excellence (NICE) CKD guidelines, CKD is the condition of multiple renal damage or drop-in glomerular filtration rate (GFR 60 ml/min per 1.73m2) for a time period of 3 months. The accessible indicator for estimation of kidney function is GFR, which equates to an aggregate sum of fluid filtered through the entirety of the working nephrons, per unit of time [4]. The preferred equation to calculate GFR is that of the CKD Epidemiology Collaboration (CKD-EPI), which takes into consideration ethnicity, age, sex, and serum creatinine concentration, whereas proteinuria is better assessed by the urinary albumin-to-creatinine proportion [17]. And on the basis of results, a 5 staged classification of CKD is made under which, proteinuria and GRF level between 60 and 90 mL/min/1.73 m^2^ leads to classification of the individual under stages 1 and 2 of CKD. This is followed by clinically significant CKD, marked as stages 3 with a GFR of 30–59 ml/min/1.73 m^2^, and stage 4 with a GFR range of 15–29 ml/min/1.73 m^2^. Furthermore, it was suggested under the NICE CKG guidelines that stage 3 should be divided into 3a (GFR 45–59 ml/min/1.73 m^2^) and 3b (30–44 ml/min/1.73 m^2^), whereas in the case of a 15 ml/min/1.73 m^2^ rate of glomerular filtration or if the individual needs to undergo dialysis, this stage 5 CKD is termed "end-stage renal disease" (ESRD) [18].

CKD has a huge economic burden on health care systems [19]. According to the US annual data report of 2020, an increase of 3.6 per cent (per 100 patients) in renal transplant was observed among patients undergoing dialysis in 2018. And until the end of 2018, there were 229,337 functional renal transplants and 554,038 patients undergoing dialysis [20]. This is the reason for which CKD was ranked 10th in India according to the CDC's (Centre of Disease Control and Prevention) report of ‘top 10 causes of death" in 2021 [21]. Further, the expense of treatment was the most noteworthy for patients in CKD stage 5 in correlation with those in CKD stages till 4, as majority of the patients in CKD 5 phase were on dialysis and needed erythropoietin additionally to alternative medicines. Unaffordability because of significant expense and lower salary of patients in lower financial status stays to be one amongst the foremost well-known explanations behind nonadherence to dialysis among patients with CKD [22].

Patients with CKD were found to have disturbances in the phosphorus, calcium, and vitamin D metabolic pathways [23]. As in some cases, due to lack of precursor as well as abnormal functioning of the renal enzyme 1-hydroxylase, patients frequently develop deficiency of active vitamin D which eventually causes hypocalcaemia and secondary followed by tertiary hyperparathyroidism leading to stimulation of bone osteoclast activity [24]. In addition to this, another possible effect of CKD is ESRD, which leads to therapies including dialysis or renal transplantation [18]. It affects 285 million adults worldwide, which has a higher probability of incrementing up to 69 per cent in developed countries and nearly equal to 20 per cent in other countries by 2030 [24].

The tools of recent science keep on divulging effective novel biomarkers that can be inserted within the kidney and show the seriousness and, progression of CKD when estimated non-invasively in urine. These incorporate LFABP (Liver-type fatty acid binding protein), NGAL and KIM-1. NGAL, or lipocalin 2 (LCN2), is a glycoprotein that is attached to matrix metalloproteinase-9 in human neutrophils and is one of the most broadly considered renal biomarkers. In this case, level of NGAL in urine has been exhibited to be inversely related with estimated glomerular filtration rate (eGFR) and straightforwardly associated with both interstitial fibrosis and cylindrical decay [25]. Likewise, various circulating proteins get accumulated in CKD and are tested as they may have the potential to be used as biomarkers of CKD progression. apoA-IV, FGF23 (Fibroblast Growth Factor-23), NGAL, adiponectin, ADMA (asymmetric dimethylarginine), and natriuretic peptides are some of those biomarkers [26].

In case of children, cause of CKD is a bit different as compared to that in adults. In a recent report from NAPRTCS (North American Paediatric Renal Trial and Collaborative Studies), congenital anomalies of the kidney and urinary tract (CAKUT) (48 per cent) and hereditary nephropathies (10 per cent) as well as glomerulonephritis account for 14 per cent of total case studies. The aforementioned causes vary with physiological factors like age; however, CAKUT is more prevalent in younger patients [27].

In developed countries, people of a higher age group and suffering from obesity and cardiovascular diseases are probable suspects for CKD, most notably type 2 diabetes and hypertension [28]. And in most of the developing countries, diabetes is found to be most common reason behind renal failure which leads to 40 per cent or more of new patients [29].

## Treatment

For people diagnosed with AKI, one needs to have a record of their renal functioning by evaluating their serum creatinine level and urine output, and then accordingly, treatment with diuretics, intravenous fluids, and/or different haemodynamic methods are prescribed [30]. Various medications and investigational compounds, after trials on various animal models, appear to be encouraging against AKI (Table 1) and are utilised in trials for a range of indications [31, 32].

**Table 1:** Emerging pharmacological agents for the treatment of AKI in various models

Drugs	Properties
Teprasiran (QPI 1002)	Teprasiran blocks the expression of p53, which promotes apoptosis [33]
Levosimendan	levosimendan increases Renal Blood Flow (RBF) through renal vasodilation with preference for the afferent arterioles, resulting in higher intra-glomerular pressure and increased filtration [34]
Calcitriol [1,25-dihydroxy vitamin D]	Within the proximal tubule of the nephron, the enzyme 1-alpha-hydroxylase transforms calcifediol into calcitriol i.e., the bioactive form of vitamin D which has shown improvement in patients with damaged kidney [32]
Alkaline phosphatase	Lipopolysaccharide, a pathogen-associated molecular pattern that causes inflammation in sepsis-associated AKI, is detoxified by the endogenous enzyme alkaline phosphatase by dephosphorylation. Moreover, recombinant alkaline phosphate also had a positive impact on long-term renal function [35]
Nitroglycerin	Nitroglycerin significantly increases the creatinine clearance levels, renal blood flow rate, and serum electrolytes (sodium and potassium) that were disrupted by ischemia–reperfusion. Additionally, it dramatically reduced the effects of a compromised antioxidant defence system and prevented lipid peroxidation [36]
Furosemide	Furosemide, loop diuretic medicine, working kidney’s helper to remove excess water and electrolyte so being utilised during emergency and when patient in Intensive care unit [37]

In general, loop diuretics were seen to be utilised in oliguric AKI and theoretically reduce the effect of renal ischemia by restricting metabolic requirements in the oxygen deprived medulla by inhibiting sodium/potassium/chloride co-transporters. Nonetheless, clinical trials have not consistently demonstrated a benefit of diuretics in AKI [30].

Despite various classes of antihypertensive agents for the CKD patients, traditional way of treatment includes Renin Angiotensin aldosterone system (RAAS) inhibitors as first preference. RAAS inhibitors not only helps in lowering blood pressure but also shows albuminuria-lowering effects [38]. In case of declination in kidney function, high dose loop diuretics are in use to control fluid retention and accompanying hypertension. Diuretics increases the efficiency of RAAS inhibitors in lowering albuminuria; results in additional renoprotection [39].

Traditional Chinese herbal medicine has been shown in numerous investigations of cell science, physiology, pathology, and modern clinical science to be an effective elective treatment for CKD. There are a few clinical reports in Chinese writings, on the beneficial impacts of various decoctions, herbs, and medicines for diuresis, hyperlipidemia, improvement of hypoalbuminemia, reducing proteinuria, and renal insufficiency in both diabetic and non-diabetic CKD. There are many Chinese herbs utilised to treat CKDs, some of which are: *Salvia miltiorrhiza*, *Astragalus membranaceus*, *Rheum officinale*, *Astragalus membranaceus*, *Plantago asiatic,* and *Salvia Miltiorrhiae* [40]. Several drugs and compounds under study in CKD studies are presented in Table 2 and are being tested for a variety of indications [41].

**Table 2:** Drugs under scrutiny for the treatment/anticipation of CKD [41]

Action/Mechanism	Drugs
Blockade of renin	Aliskiren
Inhibition of TGF-b–mediated Fibrosis	Pirfenidone
Activation of over 250 cytoprotective genes, with protective activity on immune-mediated inflammation	Bardoxolone methyl
Inhibition of CCL2 (also called MCP-1)	Bindarit
Inhibition of ET-1–mediated arterial vasoconstriction, glomerular hypertension, increased proteinuria, and interstitial fibrosis	ET-1 antagonist
Restoration of heparane sulphate component of basement membrane	Sulodexide

There are various data that support the use of several traditional Chinese medicines against AKI treatment by different approaches, such as by constraining cell apoptosis, necroptosis, ferroptosis, and inflammation, some reports claim the use of such medicine can trigger kidney damage, which eventually leads to AKI [42, 43]. Some of those medicines include aristolochic acid, anthraquinones, flavonoids, and glycosides [44]. As per some research, it was found that the application of aristolochic acid for other diseases can end up causing acute tubular necrosis, progressive tubulointerstitial injury, and ultimately renal fibrosis [44, 45]. Other similar examples include triptolide, isolated from *Tripterygium wilfordii Hook F.* (TwHF), which is generally used against cancer or as an immunosuppressant even though it can instigate AKI [44, 46]. Further, some biflavonoids, amentoflavone and sciadopitysin obtained from *Ginkgo biloba*, possess anti-inflammatory, antiviral, anti-tumoral, as well as anti-diabetic properties. But as per the findings of LI et al., these flavonoids have the potential for hepatic and renal toxicity [47]. These results could be because of numerous factors such as inaccurate dosing, adulterations, heavy metal contaminants, intrinsic toxicity, and many more. So, with acceptance of the enormous pharmacological applications of traditional Chinese herbal medicine, some work is still needed before depending completely on them. These treatments are not applicable to treat renal tissue damage and regeneration of lost tissue to retrieve proper functioning of kidney.

It has been known that kidney regeneration involves a series of activities. In the first place, it begins with the dedifferentiation and proliferation of damaged proximal tubular epithelial cells. Utilisation of fate-mapping techniques indicates that the intrinsic surviving tubular epithelial cells are dominant over new cells during repair of the post-ischemic nephron. Second, distal tubular cells can discharge development factors, such as hepatocyte growth factors (HGF). Such reparative growth factors follow up on specific sorts of cells to enhance recovery via paracrine impact [48]. Stimulation of the HGF–Met pathway induces dynamic biological responses that play important roles in the regeneration, protection, and homeostasis of cells such as hepatocytes, renal tubular cells, and neurons [49]. Also, macrophages can synthesise development factors, like Wnt7b, which enhances the multiplication of tubular epithelial cell, formation of blood vessels, and restores damaged kidney [50]. Since the last decade, several studies have been conducted in order to explore this knowledge in the field of regeneration therapy or stem cell therapy.

## Stem cell treatment

Kidney diseases fall under a heterogeneous group of disorders that affect both the morphology and the anatomical composition of human kidneys. Irrespective of general trends, some more effective techniques are now on the menu. It has been recorded that stem cells (SCs), with respect to other cells, could be used to treat damaged renal cells because of their potential to secrete growth factors or immunomodulators [51]. SCs are special cells with the potential for both multilineage differentiation and self-renewal [52]. In the zygote, which has the capacity to develop into an entire organism, stem cells first appear. Additionally, some of these totipotent zygote cells follow a particular pathway and become more constrained [53].

SCs can be categorised, on the basis of their isolation source, as adult stem cells (ASCs) and embryonic stem cells (ESCs) [54]. ASCs can then be further divided into subcategories based on their niches or action sites: including mesenchymal stem cells (MSCs), adipose stem cells (AdSCs), renal/progenitor stem cells, bone-marrow-derived stem cells (BMDSCs), and many more that could be used in vivo to treat renal damage in AKI and CKD patients. [54, 55]. Earlier, these induced Pluripotent Stem cells (iPSC) were found to be derived from fibroblast cultures by adding only c-Myc (cellular Myelocytomatosis), Klf4 (Kruppel-like factor), Oct3/4 (octamer-binding transcription factor 3/4), and Sox2 [(sex determining region Y)-box 2] [56]. Following this breakthrough, additional researchers created iPSCs using skeletal myoblasts [57, 58], peripheral blood mononuclear cells (PBMCs) [59], and other somatic cell types [58, 60, 61].

### Bone marrow-derived stem cells (BMDSCs)

Bone marrow is the niche of two different populations of SCs: Mesenchymal stem cells (MSCs) and Hematopoietic stem cells (hSCs), well proven for organ repair. It was discovered that engrafted BMDCs into the damaged kidney participate in normal tubular epithelial cell turnover as well as maintenance after AKI [62].

### Mesenchymal stem cells (MSCs)

Mesenchymal stem cells are the undifferentiated adult stem cells with bone marrow stroma as primary source apart from a number of other tissues in human body. As stated by the International Society for Cellular Therapy, these cells are known as multipotent cells with plastic-adhesive properties and can be isolated by using markers CD90+, CD105+, CD11b /CD14, CD19 /CD79a, CD34, CD45, and HLA-DR (Human Leukocyte Antigen-DR) [63].

MSCs have the potential to transform into nearly all kinds of mesodermal cell lines, including adipocytes, fibroblasts, chondrocytes, myocytes, and osteocytes [64]. Further, based on certain reports, these cells can give rise to cells of both ectodermal and endodermal origin [65]. MSCs derived from human ESCs could be utilised effectively in preclinical lupus nephritis with enhanced proliferative potential [66]. Human iPSC-derived MSCs have also been administered to CKD rat models, which successfully mobilised into kidney parenchyma and maintained the urinary clearance of creatinine [67].

MSCs promote regeneration of damaged tissues either by cell-to-cell interaction or by secreting biomaterial; antioxidants, antiapoptotics, and growth factors including epithelial growth factors, vascular endothelial growth factors, transforming growth factors, fibroblast growth factors, insulin-like growth factors type 1, and a few more are examples [68], which finally stimulate the differentiation of local progenitor cells. The therapeutic capability of MSCs is moreover identified with a potential suppressive immunological effect, that could enhance the efficiency of pharmacological prophylaxis to prevent transplant rejection [69]. Such an immunosuppressive effect nominates MSCs as a promising therapeutic candidate for graft-versus-host disease, Crohn’s disease, multiple sclerosis, and inflammatory kidney disease [70].

The effectiveness of MScs is demonstrated by a study of 30 patients, one-third of whom were renal transplant patients with heterogeneous CKD and reported improvement in kidney performance 6 months following autologous BM-MSC [71].

At the time of kidney development, there is the presence of self-renewing cells in the form of dense mesenchyme near the edge of the uretic duct, with the tendency to generate nearly all elements of the kidney, including nephrons, interstitium, and vasculature, through an initial mesenchyme-epithelial transition event. This condensed mesenchyme is termed the Renal Progenitor Cells (RPCs) or Renal Stem Cells (RSCs) population [72]. Based on their niche inside the kidney, a variety of markers could be used to identify heterogenous groups of renal stem cells. They express CD73, CD29, CD44, CD146, SSEA-4, cytokeratin, vimentin, Pax2, Six1, Six-2, cMyc, Klf4, and Oct-4 [73].

Along with their promising nature, there are some limitations associated with the use of MSCs for treatment against kidney damage. According to a research study, factors like the inflammatory environment of a damaged kidney and insufficient expression of adhesion molecules such as ICAM-1 and VCAM-1 hinder the activity of MSCs [74].

Moreover, the lack of accurate and up-to-date manufacturing protocols, along with knowledge regarding the use of higher doses/frequency and mode of administration, obstruct the implication of MSCs therapy for kidney treatment [75, 76]. Although various research supports the use of MSCs, some reports highlight the negative outcomes. In one study, syngeneic MSCs delivered to a rat model failed to prolong rat kidney graft survival, and in another study, a rat model that received syngeneic MSCs pre-transplantation, showed graft dysfunction within 7 days post-transplantation [77].

### Adipose-derived MSCs (AdMSCs)

AdMSCs are another type of stem cell with comparable properties to bone marrow derived MSCs. They can be differentiated into adipocytes, myocytes, neurons, and osteoblasts. Apart from these properties, AdMSCs are found to have more effective anti-inflammatory and immunomodulating functions as compared to MSCs [78]. Typically, liposuction is used to separate these cells from human fat [54], though their isolation is a bit complicated because of the diversified sources of adipose tissue. For example, AdMSCs obtained from the subcutaneous layer and visceral adipose tissue express higher CD10 and CD200 levels, respectively [79].

According to a report, when a group performed the insertion of hypoxia preconditioned human AdMSCs at reperfusion in a rodent model of renal ischemia reperfusion injury (IRI), they got positive results [69]. Furthermore, some studies found that these AdMSCs were effective in reducing the severity of IRI and in preventing the further spread of renal fibrosis after injury through suppressing oxidative stress and the inflammatory response [80].

### Hematopoietic stem cells (hSCs)

hSCs are derived from bone marrow, which can differentiate any type of blood cell, including myeloid as well as cells of lymphoid lineage. Aside from that, they can repair bone marrow damage caused by any disease or irradiation [81]. If these cells are inserted through intravenous injection, then they can be beneficial in order to enhance kidney repair. Repair of kidney microvasculature and tubular epithelial cells was observed when hSCs expressed markers consistent with endothelial progenitors and were injected. The proximal tubules within the outer stripe of the outer medulla are at high risk for IRI and are also liable for acute renal failure. Even in such cases, the paracrine mechanism was observed to repair the kidney after IRI [82].

Despite being rare (up to 0.005–0.01), they could be collected from the bone marrow by performing fluorescence-activated cell sorting (FACS), which is completely based on the surface markers of hSCs as well as their tendency to efflux mitochondrial dyes [14]. The theory that defines stem cells as organ-specific and lineage-restrictive was opposed after the disclosure that hematopoietic stem cells can proliferate and regenerate to form nonhematopoietic cell lines and can also regenerate a lethally irradiated tissue [83]. This multipotent property of hSCs suggests that they can differentiate themselves into renal cells at the time of renal damage. Also, hSCs could be reprogrammed as well as they retain developmental plasticity, in order to activate the genes that are essential for the cells to differentiate into specialised cells of the organ into which they are injected. Another property of hSCs that differentiates them from other types of cells includes the isolation of cells from various parts of the body, such as bone marrow, the umbilical cord, and the blood [84], which circulates throughout the body as peripheral circulation. In a report, successful induction of mouse hSCs and progenitor cells into renal cells was observed by Li et al. After 2 and 6 months of observation, tubular structure, expression of E-cadherin with other renotropic factors, and absence of teratoma indicated effective production of renal-like cells [85].

In another report, when cord blood cells (CD34+) are moved to the damaged renal area, it promotes survival, enhances restoration of the damaged kidney, and facilitates recovery of renal tissues by paracrine systems coordinated at peritubular vessels. These discoveries strengthen the role of cord blood cells as a potent restorative procedure for therapeutic use against AKI [84]. According to a study, hSCs from male Rosa26 mice, which expressed galactosidase, were injected into female mice suffering from I/R injury. As a result of the administration of X-Gal staining, galactosidase-positive cells were detected after four weeks of experimentation in the renal tubules of the recipient kidney [14].

Then again, it has become almost certain that endothelial progenitor cells (EPCs) in blood might help in vascular repair [86]. It was demonstrated that mononuclear blood cells with CD34 and Flk-1 markers can accept an endothelial phenotype in vitro, which was exhibited by their expression of CD31, E-selectin, Flk-1, cNOS, and Ulex europaeus agglutinin. An exceptionally late investigation portrayed a group of CD14+/CD34 cells as a significant source of circulating progenitors [87].

### Embryonic stem cells (ESCs)

ESCs are collected from the embryo before its implantation inside the uterus during its developmental stage. This 32-cell stage is known as the blastocyst stage, through which isolation of such pluripotent cells can be done [88]. ESCs have the ability to generate in all three embryonic germ layers. As the work on ESCs showed, these cells can be proliferated to get renal cells and can work more specifically after their differentiation into various kidney organoids, such as glomerular-like structures or renal proximal tubules [89, 90]. There are several proteins that govern ESC pluripotency, including the Oct 4 protein and the Nanog protein. Some studies incorporated the Wnt-catenin signalling pathway, which is responsible for maintaining pluripotency, as a continuation of earlier works [91].

These mouse ESCs were stimulated to develop into cells that express the distinctive marks of both mesoderm and renal embryonic precursors by administering retinoic acid and Bmp7 (Bone Morphogenetic Proteins) [5]. Recently, several groups have worked towards the production of high-performance renal organoids. Multiple protocols for the generation of various renal cell types had now been introduced into the picture. There have also been reports of the induction of nephron progenitors into nephron-like structures [92–94]**.**

In order to successfully produce and assemble renal lineage cells, Taguchi et al. used both mouse embryonic stem cells and human induced pluripotent stem cells [95]. In addition, Tanigawa et al. proposed an in vitro procedure in 2022 that would use mouse ESCs to create stromal progenitors that would eventually develop a complex kidney structure [96]. As per the protocol exercised by Takasato and colleagues, nephrons connected to a network of collecting ducts and encircled by renal interstitium and endothelial cells were found in developed kidney organoids. They observed that individual nephrons divide inside these organoids to form distal as well as proximal tubules, loops of Henle, and glomeruli that contain podocytes going through vascularization. Further, human foetal tissues and kidney organoids' transcription patterns were examined, and the first trimester of the human pregnancy was found to have the highest concordance [97].

In one of these studies, they compared the induction of wild type nephron progenitor cells and mutant type nephron progenitor cells, both derived from mouse embryonic cells. The modified approach improved the expression of significant NPCs markers such as Cited2, Wt1, Hoxd11, and Six2. And further, the approach claimed the potential of derived wild type progenitor cells to develop interactions with functional UB and possess a tendency to undergo nephrogenesis [98]. Chow T et al. proposed a protocol for differentiating mouse embryonic stem cells into renal progenitor cells in 2020. They tried to mimic the embryonic kidney development in mesoderm, intermediate mesoderm, and renal progenitors so that eventually kidney organoid synthesis could be achieved. In the end, various renal progenitors, including nephron progenitors, ureteric bud cells, and stromal progenitors, were obtained. However, the protocol was limited by the fact that it took significantly longer for the kidney to mature than a mouse's usual gestation period, which was explained by missing signalling molecules in the culture environment [99].

Hence, there is a requirement for better strategies for the isolation of pure renal cells; otherwise, in vivo undifferentiated embryonic stem cells may result in teratoma [100]. Also, there are some ethical issues with the use, generation, and isolation of human ES cells. Producing autologous hESCs without somatic cell nuclear transfer is restricted by the legal prohibition of genomic-imprint reprogramming, so for stem cell therapy, cells must be collected from an allogenic source, albeit a few articles have recommended the immunological benefits of ESCs [101].

### Induced pluripotent stem cells (iPSCs)

Successful reconstruction of fibroblasts into ESC-like cells by ectopic expression of just a bunch of ‘stemness’ factors as well as expectations of the potential home of therapeutic cells for personalised tissue repair are named "induced pluripotent stem cells" (iPSCs) [102]. These iPSCs are like ESCs in that, in suitable culture conditions, they can be differentiated indefinitely to maintain pluripotency as well as be induced to differentiate into all derivatives of the three primary germ layers: ectoderm, endoderm, and mesoderm [103]. However, like ESCs, the pluripotency of iPSCs does have some post-transplantation limitations if the cells are directed without pre-differentiation. In such a case, there are 20 per cent chances that the offspring may eventually end up developing tumours [104].

Although being significant developmental milestones in the field of stem cell therapy and regenerative medicine, due to tumorigenicity, immunogenicity as well as heterogeneity induces some restriction over large-scale applications of iPSCs. Thus, recent progress shifted towards the generation of iPSCs from other somatic cells, including human breast milk epithelial cells [44], epidermal keratinocytes [77]**,** ligament cells [76]**,** skeletal myoblasts [98, 99]**,** synovial cells [105], and peripheral blood mononuclear cells [106, 107]**.** Moreover, several approaches are now being implemented in order to prevent such risks.

Furthermore, the Sendai virus approach, which includes single-strand RNA replication in the cytoplasm without genome integration, avoids the limitations of commonly used integrating viral vectors, such as lentivirus [108]. Moreover, PiggyBac and Sleeping Beauty are among the direct and promising transposon approaches with stable expression of reprogramming factors as well as safe and cost-effective alternates to viral delivery methods [109]. In support of this, Vanslambrouck et al. developed an improved procedure for the direct reprogramming of human kidney 2 (HK2) and human renal epithelial cells for the production of induced nephron progenitor-like cells using SNAI2, EYA1, and SIX1 transcriptional factors [110].c-Myc is the most controversial, and hence multiple combinations of transcriptional factors are under trial. Several studies claim enhanced potency for renal repair and regeneration without c-Myc factor [111, 112]. Supporting this, Morizane et al. [113] reported the production of kidney organoids, mostly by the stimulation of CHIR and FGF9 in a 3D culture. Initially, they differentiated human iPSC into primitive streak cells, followed by induction into posterior intermediate mesoderm and nephron progenitor cells. Toyohara et al. [114] made a significant contribution to the treatment of AKI by developing a multi-step differentiation protocol in which hiPSCs were induced to transform into renal progenitor cell with marker Odd-skipped related 1 (OSR1) and SIX2. In tests conducted both in vitro and in vivo, differentiated renal progenitor has demonstrated its capacity to remodel proximal renal tubule-like shape. Amelioration of AKI has been observed when OSR1+(Odd-skipped related 1) SIX2+ renal progenitor was transplanted in the mice. Further, Hoshina et al. [115] described the improved renal function and reduced tissue damage, indicated by decreased fibrosis, tubular dilatation, and loss of tubular borders by iPSC-derived renal progenitor cells (CD9− CD140a+ CD140b+ CD271+ cells) against AKI.

Furthermore, the potency of iPSC could be explained in more studies, such as how undifferentiated murine iPSCs, when administered through the intraarterial route, attenuate ischemia reperfusion-induced AKI mediated through paracrine action [116]. As indicated by Lazzeri et al. [117], progenitor cells of a specific tissue have the potential to cure tissue-specific diseases, which could be acquired by the differentiation of embryonic or induced pluripotent cells. Still, continuous work is required in order to standardise a step-by-step protocol in such a way that such stem cells should initially form intermediate mesoderm, followed by the development of progenitor cells and other renal cells. hPSCs (including both human iPSCs and human ESCs) were successfully directed to form kidney organoids by inducing four progenitors: nephron progenitors, renal interstitial progenitors, endothelial progenitors, and uretic epithelial progenitors. In formed organoids, segmented nephrons are linked with collecting ducts in the surrounding renal interstitial cells and a network of endothelial cells [118].

Also, they should be trained to overcome present issues, including heterogeneousness, differentiation potential, interline changeability, and inefficient transduction in induced pluripotent cells [117].

BMSCs and iPSs were effective in improving renal capacity since they expanded the CCr (creatinine clearance), attenuated the elevation in serum creatinine, and hindered the RCCr (Rate of decline in the CCr) at the end of the examination time frame [119].

### Amniotic fluid derived stem cells (AFSCs)

AFSCs are another type of SC whose characteristics lie somewhere between those of embryonic and adult SCs. They can be transformed into renal cell lines while retaining the properties of epithelial as well as podocyte markers when refined in specific culture conditions [120]. Flow cytometry results of AFSCs showed CD73, CD90, and CD105 MSC markers as well as CD14, CD20, CD34, and CD45 hSC markers [121]. When human AFSCs were injected into the kidney after a dose of glycerol injection, a positive result was observed in the levels of blood urea nitrogen (BUN) as well as in serum creatinine levels. They do have immunomodulatory effects, which were found to manage immunological response in support of tissue cytokines and the cellular environment to prevent or restore damaged tissues. During working with these cells in an acute tubular necrosis mouse model, Hauser et al., found that injection of human AFSCs improved the rate of creatinine reduction, BUN level, apoptosis reduction, and promotion of tubular cell proliferation [122]. And in support of the above study, AFSCs with SIRT3 (suppresses hypoxia-inducible factor 1) were found to increase engraftment and promote differentiation into endothelial cell lineages when transfected with GDNF (Glial Cell Line Derived Neurotrophic Factor) [123].

On correlating the property of AFSCs of regeneration in the AKI model with that of MSCs it was found that MSCs were more productive in inciting expansion. And in a cisplatin-injured kidney, Rota et al. demonstrated that human AFSCs improve renal functions as well as limit tubular damage by activating local paracrine signals instead of cell proliferation and differentiation [124].

## Conclusion

Kidney disease and failure are raising the financial burden as well as contributing to an increase in the number of deaths due to health diseases, and such statistics are rising each year. The use of cell therapy in several research studies has shown promising results over conventional medicine and treatment in restoring or at least improving kidney functions in preclinical models of AKI and CKD. Because of the distinct characteristics of various types of stem cells, they are a viable candidate for treating various kidney injuries. MSCs could be the most promising candidates for autoimmune-related kidney disease because of their immunomodulatory properties. AdMSCs have demonstrated their ability to treat renal fibrosis and lessen the severity of IRI. hSCs, on the other hand, can be used to improve damaged renal functions by providing paracrine growth factors to the damaged renal cells. *ESCs were successfully differentiated into stromal progenitors, which eventually developed a complex kidney structure and played an important role in nephrogenesis.* However, there is a limitation to the refined protocol required to extract its potential and regenerate renal tissues. iPSCs ignite the new hope in stem cell research because they can be engineered from adult tissues and have properties like ESCs. But there are some limitations in their developmental protocol, which is being modified by using new methods like the Sendai virus, PiggyBac, and Sleeping Beauty approach. By activating local paracrine signals, human AFSCs enhance renal functions and reduce tubular damage. In certain studies, blood creatinine and BUN levels also decrease following an AFSC injection. Kidney organoids from hPSC also provided hope for renal tissue regeneration. Overall, SC has potential in the regime of renal tissue repair and regeneration, but still, there are some challenges, including laboratory expenses, heterogeneity of kidney patients as well as the stem cell population. But their limitations could be overcome, with more knowledge regarding the in vivo mechanisms of various stem cells, their sources, dosage, and route of administration.

## Data Availability

Not applicable.

## Abbreviations

GFR: Glomerular filtration rate
AKI: Acute kidney injury
CKD: Chronic kidney disease
IL-18: Interleukin-18
NGAL: Neutrophil gelatinase–related lipocalin
KIM-1: Kidney Injury Molecule-1
COVID-19: Coronavirus disease 2019
ACE-2: Angiotensin converting enzyme 2
IRI: Ischemia–reperfusion injury
NKF-KDOQI: National Kidney Foundation-Kidney Disease Outcomes Quality Initiative
NICE: National Institute for Health and Care Excellence
CKD-EPI: Chronic kidney disease-epidemiology collaboration
ESRD: End-stage renal disease
CDC: Centre of Disease Control and Prevention
L-FABP: Liver-type fatty acid binding protein
LCN-2: Lipocalin 2
eGFR: Estimated glomerular filtration rate
FGF-23: Fibroblast growth factor-23
ADMA: Asymmetric dimethylarginine
NAPRTCS: North American Paediatric Renal Trial and Collaborative Studies
CAKUT: Congenital Anomalies of the Kidney and Urinary Tract
BMDC: Bone marrow-derived cell
ECOS: Extracorporeal organ support
RAAS: Renin Angiotensin Aldosterone System
SCs: Stem cells
ESCs: Embryonic stem cells
ASCs: Adult stem cells
MSCs: Mesenchymal Stem Cells
AdSCs: Adipose stem cells
BMDSCs: Bone-marrow-derived stem cells
c-Myc: Cellular Myelocytomatosis
Klf4: Kruppel-like factor
Oct3/4: Octamer-binding transcription factor 3/4
Sox2: (Sex Determining Region Y)-box 2
hSCs: Hematopoietic stem cells
RPCs: Renal progenitor cells
RSCs: Renal stem cells
VEGF: Vascular endothelial growth factor
AdMSCs: Adipose-derived mesenchymal stem cells
FACS: Fluorescence-activated cell sorting
IGF1: Insulin-like growth factor 1
G-CSF: Granulocyte-colony stimulating factor
EPCs: Endothelial progenitor cells
Bmp7: Bone Morphogenetic Proteins 7
iPSCs: Induced pluripotent stem cells
CCr: Creatinine clearance
RCCr: Renal creatinine clearance
BUN: Blood urea nitrogen
GDNF: Glial Cell Line Derived Neurotrophic Factor
